# Nucleation as a rate-determining step in catalytic gas generation reactions from liquid phase systems

**DOI:** 10.1126/sciadv.ade3262

**Published:** 2022-11-16

**Authors:** Thomas Solymosi, Michael Geißelbrecht, Sophie Mayer, Michael Auer, Peter Leicht, Markus Terlinden, Paolo Malgaretti, Andreas Bösmann, Patrick Preuster, Jens Harting, Matthias Thommes, Nicolas Vogel, Peter Wasserscheid

**Affiliations:** ^1^ Helmholtz-Institute Erlangen-Nürnberg for Renewable Energy, Forschungszentrum Jülich, 91058 Erlangen, Germany.; ^2^ Department of Chemical and Biological Engineering, Friedrich-Alexander-Universität Erlangen-Nürnberg, 91058 Erlangen, Germany.

## Abstract

The observable reaction rate of heterogeneously catalyzed reactions is known to be limited either by the intrinsic kinetics of the catalytic transformation or by the rate of pore and/or film diffusion. Here, we show that in gas generation reactions from liquid reactants, the nucleation of gas bubbles in the catalyst pore structure represents an additional important rate-limiting step. This is highlighted for the example of catalytic hydrogen release from the liquid organic hydrogen carrier compound perhydro-dibenzyltoluene. A nucleation-inhibited catalytic system produces only dissolved hydrogen with fast saturation of the fluid phase around the active site, while bubble formation enhances mass transfer by more than a factor of 50 in an oscillating reaction regime. Nucleation can be efficiently triggered not only by temperature changes and catalyst surface modification but also by a mechanical stimulus. Our work sheds new light on performance-limiting factors in reactions that are of highest relevance for the future green hydrogen economy.

## Teaser

Nucleation is an overlooked critical rate-determining step in catalytic gas generation reactions from liquid phase systems.

## INTRODUCTION

Catalysis is of utmost importance for the production of chemicals and for many forms of chemical energy storage. It is assumed that about 90% of all chemical processes proceed with the aid of at least one catalyst, where 80% use solid, heterogeneous catalysts (*1*). It is known that the observable reaction rate of heterogeneously catalyzed reactions is limited either by their chemical kinetics or by the transport rate of the reactants, either within the porous structure of the catalyst (pore diffusion limitation) or from the bulk phase to the surface of the catalyst pellet (film diffusion limitation). Here, we report an additional, previously overlooked limitation that occurs in heterogeneously catalyzed liquid phase reactions that produce a large amount of gaseous products. This additional limitation is determined by the ability of the catalytic interface to nucleate product gas bubbles, a factor that has been found in our experiments to vary the productivity of a given catalyst system under identical reaction conditions by more than a factor of 50. In this contribution, we exemplify the relevance of nucleation on the reaction rate of catalytic gas formation reactions for hydrogen release from the liquid organic hydrogen carrier (LOHC) perhydro-dibenzyltoluene (H18-DBT), a highly relevant reaction for future hydrogen storage and transport technologies (*2*, *3*).

Nucleation is driven by the supersaturation of a dissolved gas in liquid but is often hindered by the presence of an energy barrier associated with the creation of new interfaces. The nucleation process can be either homogeneous, if no solid surface is present, or heterogeneous with bubbles nucleating at a solid surface (*4*). While in homogeneous nucleation, a theoretical supersaturation of over 100 is required (*5*, *6*), a solid surface can reduce the activation energy for bubble nucleation substantially depending on its wettability and structure (*7*). Surfaces on which the used liquid medium exhibits nonwetting behavior and geometrical defects facilitate nucleation (*8*). Furthermore, bubble growth can occur from a new nucleus or from preexisting gas in the catalyst pores.

The relevance of bubble nucleation for the rate of relevant multiphase processes has been recognized for a couple of technically relevant applications but, so far, not for classical heterogeneous catalysis. In contrast, the gas evolution rate of different electrochemical processes, including hydrogen production in water electrolysis (*8*, *9*), gas evolution in aluminum electrolysis (*10*), and CO_2_ formation in direct methanol fuel cells (*11*), has been related to the systems’ ability to nucleate product gas bubbles. Moreover, it has been found that structured surfaces enhancing heterogeneous nucleation provide positive rate effects on the oxygen evolution from hydrogen peroxide (*12*) and on boiling processes in heat exchange devices (*13*, *14*).

For water electrolysis, which is currently the best investigated example of a nucleation-influenced electrocatalytic process, it has been found that bubble nucleation is driven by the supersaturation of dissolved oxygen. Nucleation only occurs if there is transport of reactants, an electron conductor together with a catalyst to oxidize the water, and an electrolyte to transport the protons (*15*). Thus, nucleation takes place at the interface between the active electrocatalyst and the porous liquid-gas diffusion layer (LGDL). Systematic studies have shown that increasing porosities and decreasing pore sizes provide more nucleation sites at the triple-phase boundary and, thus, enhance performance (*16*). Another reported way to facilitate nucleation is to reduce water wettability on the surface, e.g., by adding a hydrophobic layer between the electrocatalyst and the LGDL (*17*, *18*). This leads to higher contact angles and to an increased nucleation rate at lower current densities.

Given these very relevant effects, it is unexpected that, to our knowledge, bubble nucleation has not yet been considered as a rate- and performance-limiting factor in thermally activated catalytic transformations using active metal nanoparticles on pelletized, porous supports. In this contribution, we demonstrate for the example of the catalytic dehydrogenation of H18-DBT that bubble nucleation represents a so-far overlooked and very relevant effect. We show that, for a catalyst consisting of platinum nanoparticles on porous alumina, conditions favoring nucleation increase the hydrogen release rate by a factor of over 50 as compared to the same catalyst material under otherwise identical conditions but in the absence of effective bubble nucleation. This mechanism adds a fundamental aspect to the relevant mass transfer limitations in heterogeneous catalysis.

## RESULTS AND DISCUSSION

### Two activation states of the same catalyst material under identical conditions

**Fig. 1.**
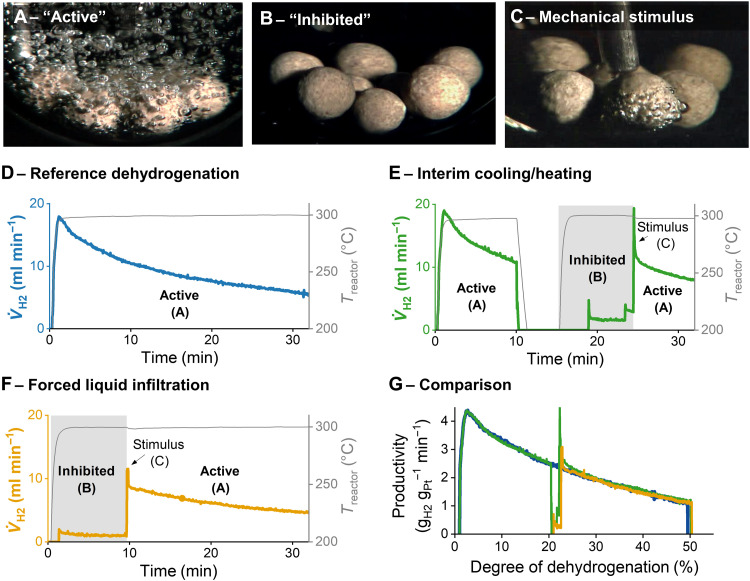
Batch dehydrogenations of 1 g of H18-DBT with eight pellets of 0.3 wt% of Pt/alumina catalyst in a glass tube heated with an oil bath show different activity depending on catalyst state. (**A**) Virgin catalyst in its active state at an oil bath temperature of 300°C. (**B**) Catalyst in its inhibited state after cooling to below 100°C and reheating to 300°C. (**C**) Activation of one individual pellet by a mechanical stimulus (movie S1). (**D**) Hydrogen release and reactor temperature over time for a standard reference dehydrogenation experiment using virgin catalyst pellets. (**E**) Generation of the inhibited state by cooling to below 100°C after 10 min and reheating to 300°C after 18 min followed by activation of each inhibited pellet by a mechanical stimulus 9 min after reheating. (**F**) A pretreated liquid-filled catalyst [liquid has 20% degree of dehydrogenation (DoDH) to mimic the experiment shown in (E)] was used as starting material for mechanical reactivation after 10 min. (**G**) Comparison of hydrogen productivity as function of the DoDH for the applied catalyst in its active state (blue), in its inhibited state through cooling and reheating (green), and in its inhibited state through liquid infiltration (orange) before and after mechanical activation.

Our experimental studies deal with the release of hydrogen from H18-DBT in a catalytic dehydrogenation reaction using a heterogeneous eggshell catalyst with 0.3 weight % (wt%) of platinum on porous alumina . These studies unexpectedly reveal that the catalytic activity of the system varies by more than a factor of 50, depending on the way the catalytic experiment is started. When a virgin catalyst material (material that has never been in touch with H18-DBT before) is contacted with hot, liquid H18-DBT [oil bath temperature: 300°C; boiling point (H18-DBT): 360°C], the hydrogen is immediately released, and bubbles turbulently burst out of the catalyst pores as shown in Fig. 1A. As long as the system is kept at this reaction temperature, the hydrogen release only slows down because of the decreasing hydrogen loading of the liquid hydrogen carrier.

A totally different picture is found when the same catalyst material is added to hot H18-DBT and the mixture is cooled down to below 100°C followed by reheating to a reaction temperature of 300°C. In this case, only less than 1.8% of the original hydrogen release activity is observed despite the fact that the hydrogen content bound to the organic carrier is still 80%. Visual inspection shows that the catalyst pellets do not form gaseous hydrogen bubbles (see Fig. 1B). Only very few bubbles occasionally form at contact points of catalyst pellets and the glass wall of the reaction vessel.

Consequently, the same catalyst exists in two different states under identical reaction conditions, depending on how these conditions have been reached. Both states, to which we refer here as “active” and “inhibited,” markedly differ by the visible bubble formation and by their productivity in hydrogen release. To our surprise, we incidentally realized that individual pellets of an inhibited catalyst system can be turned into an active state by a mechanical stimulus. Figure 1C shows the result of a mechanical impact on a previously inhibited pellet (see also movie S1). Bubbles immediately form at the contact point between the pellet and the glass wall, and within a few milliseconds, gas bubbles vigorously emerge from the entire pellet. Note that the motion of the bubbles formed at the activated pellet is under the applied conditions not sufficient to reactivate the other pellets. The intense bubbling continues after a single activation of each pellet for many hours as long as chemically bound hydrogen can be released from the liquid.

To quantify the hydrogen release rate of the different states, we carried out dehydrogenation experiments on a small scale to avoid influences of inhomogeneous heating and cooling. Only 1 g of H18-DBT and eight catalyst pellets (total catalyst mass: 111 to 129 mg) were used. Figure 1D shows the hydrogen volume flow at 300°C produced by the virgin catalyst in its active state. While the reaction system heats up, the hydrogen flow increases and reaches a maximum shortly after. The following decrease in produced hydrogen volume flow reflects the dehydrogenation kinetics: The dehydrogenation reaction slows down with an increasing degree of dehydrogenation (DoDH), indicating a positive reaction order of the hydrogen-loaded carrier H18-DBT. The experiment shown in Fig. 1E was started in the same manner, but after 10 min of runtime, when the DoDH reached 20%, the glass reactor tube was removed from the oil bath and allowed to cool down to below 100°C. When the reactor was subsequently reheated to 300°C, the catalyst was reproducibly found in its inhibited state, and the released hydrogen flow was below our detection limit of 0.2 ml min^−1^, indicating an activity of less than 1.8% compared to the active state of the same catalyst under the same conditions. After 4 min and after 9 min, two individual pellets reactivated spontaneously, resulting in an increase of activity to 29% of the reference. The other six pellets were reactivated mechanically after 25 min, and 100% of the expected activity (compared to the reference virgin catalyst at the same DoDH) was reestablished.

We now explain the unexpected inhibition and reactivation effects. First, high-speed camera observations indicated that the surface pore system of the pellet is filled with liquid in the inhibited state (see movie S2). To mimic such a condition, we immersed virgin catalyst pellets in Hx-DBT with a DoDH of 20% at 60°C under vacuum for 3 days to guarantee that all catalyst pores were entirely filled with the liquid. From Fig. 1F, it is apparent that these pellets show very similar behavior in the dehydrogenation experiment when compared to the inhibited pellets from Fig. 1E. They can also be fully mechanically reactivated. Figure 1G shows that these liquid-infiltrated catalyst pellets show even quantitatively the same type of inhibition and reactivation behavior as the catalyst inhibited by the simple thermal cycling as demonstrated in Fig. 1E. Once more, it is evident that the inhibited catalyst state induced either by the cooling cycle or by forced liquid infiltration is much less productive in hydrogen release and that mechanical activation leads to a marked increase in gas formation productivity.

### Bubble nucleation as rate-limiting step

**Fig. 2.**
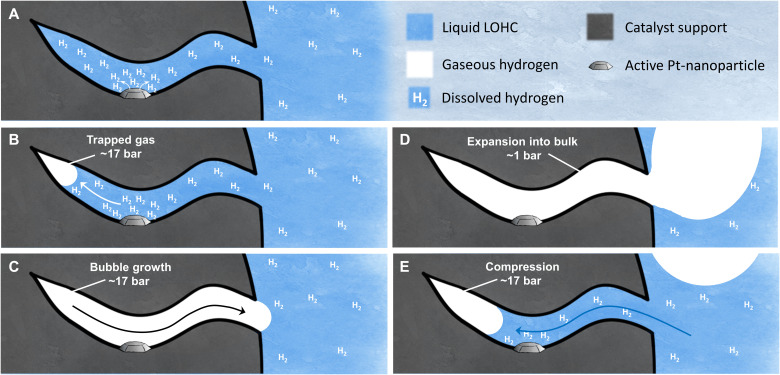
Schematic view of the inhibited and the active state of the hydrogen release catalyst. (**A**) Inhibited state without bubble nucleation, diffusive transport of dissolved H_2_. (**B** to **E**) Active state with bubble formation and different oscillating states of convective transport.

From these findings, we hypothesize that bubble nucleation within the pores may be the critical factor for the inhibition of catalytic hydrogen release in an entirely liquid-filled catalyst pore. In the inhibited state of the catalyst, no bubbles form in the dehydrogenation process (see Fig. 2A). The energy barrier associated with bubble nucleation leads to a supersaturation of the liquid with dissolved hydrogen that greatly reduces the driving force for hydrogen formation at the catalyst active site. Consequently, the diffusion of dissolved hydrogen into the bulk phase (driven by the hydrogen concentration gradient in the system) limits the overall hydrogen formation. In the active state of the catalyst system, in contrast, at least one gas bubble is present in the interconnected porous network of the pellet. These preexisting gas bubbles circumvent the necessity of a nucleation event, and hydrogen evolution can proceed directly from growth of these bubbles without the energy effort associated with creating new interfaces.

Figure 2 (B to E) illustrates the microscopic interpretation of the active state of our dehydrogenation reaction. When a catalyst pellet that is partly filled with gas is surrounded by liquid H18-DBT, the gas residue is trapped in the pores, as depicted in Fig. 2B. The gas residue is compressed because of the capillary pressure of the liquid in the pore. The resulting pressure can be estimated with Eq. 1 according to (*19*)

$${\;p}_{gas}=\frac{2\cdot \gamma (T)\cdot \cos(\theta )}{r_{\;pore}}+{\;p}_{reactor}$$ (1)

Here, *p*_gas_ is the pressure in the gas, γ(*T*) is the surface tension of the liquid, θ is the contact angle of the liquid, *r*_*pore*_ is the pore radius, and *p*_*reactor*_ is the bulk pressure. We have determined the surface tension of H18-DBT at 300°C to be 12.3 mN m^−1^. The liquid wets the alumina surface very well and shows a contact angle of <10°. With the most frequent pore radius (mode radius) being 15 nm and a reactor pressure of 1 bar in our system, the pressure in the gas can be calculated to be approximately 17 bar (details in figs. S1 to S5).

During dehydrogenation, hydrogen forms at the catalytic sites. As soon as the saturation concentration of the dissolved hydrogen is exceeded, the bubble inside the pore grows, as excess hydrogen molecules can directly translocate into the existing gas phase (Fig. 2C). When the bubble reaches the mouth of the pore, capillary effects vanish, and suddenly, much less pressure is acting on the gas, so it expands into the bulk at approximately 1 bar (Fig. 2D). Once a certain bubble size is reached, the bubble detaches. New bulk H18-DBT flows into the pore and forms hydrogen again, leading to an oscillating system with convective transport of gas and liquid in the pore.

It has been shown that, for exothermic reactions, evaporating reactants in the porous network of a liquid-filled heterogeneous catalyst can cause oscillating systems that show a 30-fold increase in reaction rate as compared to a purely diffusive mass transfer of the reactants (*20*). Rate enhancement by oscillating gas-liquid transport in porous systems was also described for the exothermal decomposition of H_2_O_2_ in a porous Ni and CuCr catalyst (*21*, *22*). We therefore interpret the enormous difference between the inhibited and the active state in our endothermic dehydrogenation reaction by the difference in mass transfer between the comparatively slow hydrogen diffusion in the liquid phase and the highly efficient mass transfer in the bubble-forming and oscillating reaction system.

The mechanism highlighted in Fig. 2 also supports our experimental finding that a virgin catalyst is always in its active state when applied in the H18-DBT dehydrogenation, as its gas-filled pore system provides an initial trigger for oscillation. Catalyst material cooled from an active process (as in Fig. 1E), in contrast, is characterized by a fully liquid-filled pore system (similar to the catalyst material obtained by forced liquid infiltration; Fig. 1F). When cooling down the system, the remaining gas bubbles in the pores are consumed because, at low temperatures, the dehydrogenation process turns into a hydrogenation process for thermodynamic reasons (*23*, *24*). Note that activity effects induced by cooling down and starting up cycles are technically extremely relevant as they form the core of all dynamic operations of these gas generation reactions, e.g., to provide hydrogen to a dynamically operating user.

To reactivate an inhibited catalyst after a cooling down and starting up cycle, it is critical to induce bubble formation. The basic prerequisite for bubble nucleation is that the saturation concentration of dissolved hydrogen is exceeded. Moreover, the new interface formation requires an activation energy to overcome a critical bubble radius. For smaller radii, the Laplace pressure is extremely high, causing the freshly formed nanobubble to dissolve again. Only when the bubble radius exceeds a critical value, further bubble growth is energetically favored. Higher supersaturation of the dissolved gas reduces the critical radius and, thus, the energy barrier, so bubble nucleation is facilitated (*25*).

In heterogeneous nucleation at solid surfaces, less new interface is created, which reduces the energy required. The reduction of the energy barrier depends on the contact angle of the liquid (*26*). A higher liquid contact angle, which is indicative of only partial wetting of the liquid, leads to a lower energy barrier for gas bubble nucleation. If, in contrast, the liquid contact angle is close to zero (complete wetting), then the energy barrier is as high as in the case of homogeneous nucleation. For a system with fixed liquid, gas, and temperature, only the surface energy of the solid affects the contact angle. Lower surface energies decrease wettability with the liquid, thus reducing the energy barrier for bubble nucleation.

**Fig. 3.**
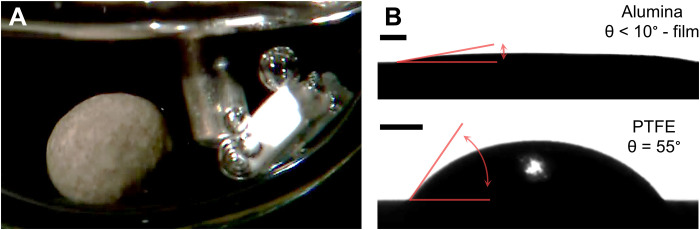
Gas nucleation on the inert PTFE cube shows the presence of supersaturated hydrogen in the bulk liquid and indicates hindered nucleation at the Pt on alumina heterogeneous catalyst pellet. (**A**) H18-DBT (0.5 g), catalyst pellet, and PTFE cube after cooling to 100°C (at 20% DoDH) and reheating to 300°C. Bubble nucleation is only observed on the PTFE surface. (**B**) Contact angle of a 3-μl drop of H18-DBT on alumina (top) and PTFE (bottom) at 300°C and H_2_ atmosphere. Scale bars, 500 μm.

To demonstrate that hindered bubble nucleation is responsible for the inhibited state of our dehydrogenation catalyst, we performed an experiment with an inhibited catalyst pellet and added a metal-free, nonporous polytetrafluoroethylene (PTFE) cube with a very low surface energy to the reaction mixture. Figure 3A shows that the hydrogen-supersaturated liquid induces hydrogen nucleation on the PTFE surface. In contrast, no bubbles are observed on the alumina surface of the catalyst pellet, where the hydrogen forms at the supported Pt nanoparticles (also see movie S3). The different nucleation efficiency can be rationalized from the involved contact angles on both solids (Fig. 3B). Under operation conditions (300°C), H18-DBT excellently wets dense alumina (top), with a contact angle of <10°, while it forms a contact angle of 55° on PTFE (bottom). Consequently, the nucleation energy barrier is much lower on PTFE, and bubbles nucleate more readily there in the presence of the supersaturated hydrogen, regardless of the fact that the hydrogen is formed on the alumina-supported Pt catalyst next to it.

### Mechanical activation of an inhibited catalyst

As shown in Fig. 1C, it is possible to mechanically reactivate the inhibited dehydrogenation catalyst to full activity (movie S4). The same activation is possible by vigorous stirring or by shaking the whole glass tube if each pellet is subjected to a critical mechanical stimulus. This critical stimulus was experimentally determined to be ≥120 nJ mm^−3^. These values are compatible with the required energy densities calculated from our bubble nucleation model that also includes the influence of the pore size of the examined catalyst pellet (further details in fig. S8).

The fact that a mechanical stimulus can serve as an energy input to overcome nucleation barriers is known from different phase transition processes: The crystallization of supercooled liquids has been triggered by mechanical pulses (*27*–*29*), crystallization of supersaturated solutions can be initiated by stirring (*30*) or by a mechanical stimulus (*31*), degassing of liquids is known to benefit from ultrasonification (*32*), and the boiling of overheated liquids is facilitated by stirring (*32*) or by vibration (*33*). While the physical basis of these processes is still under debate, local cavitation due to pressure fluctuations seems to be responsible. However, to our knowledge, the activation of a nucleation-inhibited catalytic reaction by a mechanical trigger has not been described to date.

It is a noteworthy observation from Fig. 1 (E and F) that the gas generation productivity increases sharply after the mechanically induced nucleation for approximately 30 s. This overshoot can be explained by the hydrogen supersaturation in the pore system that leads to an increased bubble formation just after the mechanical activation where many nucleation sites are created. In the course of reaction, a reduced supersaturation is reached in the activated system, and the gas generation productivity settles to the corresponding productivity of the active reference dehydrogenation.

### Strategies to avoid nucleation inhibition by chemical surface modification

Building on the observed nucleation on PTFE, we devised a surface chemistry approach to overcome nucleation inhibition. To this end, we reduced the surface energy of the alumina support by treatment with perfluorooctyl silane, hypothesizing that this surface functionality would improve bubble nucleation by reducing the wettability with H18-DBT.

**Fig. 4.**
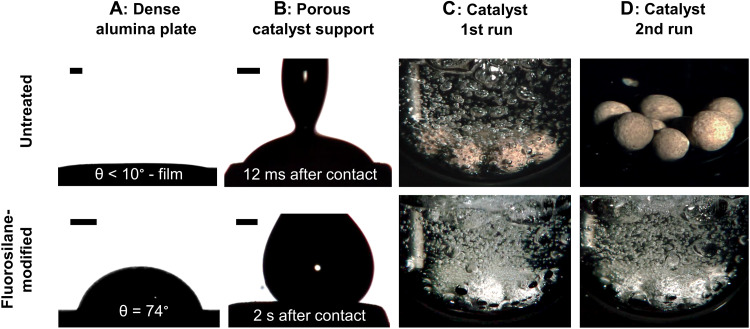
Wetting and dehydrogenation performance of alumina and catalyst samples (top: untreated) and the effect of surface modification with trichloro-perfluorooctyl silane (bottom: fluorosilane-modified). (**A**) Contact angle of 3 μl of H18-DBT on a dense alumina plate at 300°C and H_2_ atmosphere. Scale bars, 500 μm. (**B**) Contact angle of a H18-DBT droplet on the flattened porous support at 300°C and H_2_ atmosphere at different time frames. Scale bars, 500 μm. (**C**) Eight virgin catalyst pellets in H18-DBT at 300°C shortly after heating. (**D**) Eight catalyst pellets in H18-DBT at 300°C after cooling to below 100°C at 20% DoDH and reheating.

Figure 4 compares the wetting of an untreated (top) and fluorosilane-modified (bottom) alumina surface and their ability to nucleate hydrogen gas in the reaction. Whereas an untreated, flat alumina sample shows excellent wetting (contact angle of <10°), the H18-DBT droplet on the modified plate forms a contact angle of 74°, reflecting a decrease in surface energy to 14 mN m^−1^ (details in figs. S5 and S6). A more pronounced effect in wettability was observed directly on the porous catalyst pellets flattened at the top to allow droplet deposition, shown in Fig. 4B. For the untreated porous alumina pellet, the droplet immediately spread across the surface after contact, as expected for a Wenzel wetting state (*34*). In contrast, the fluorosilane-modified sample exhibited a pronounced oleophobic character, evidenced by a contact angle of 121°.

**Fig. 5.**
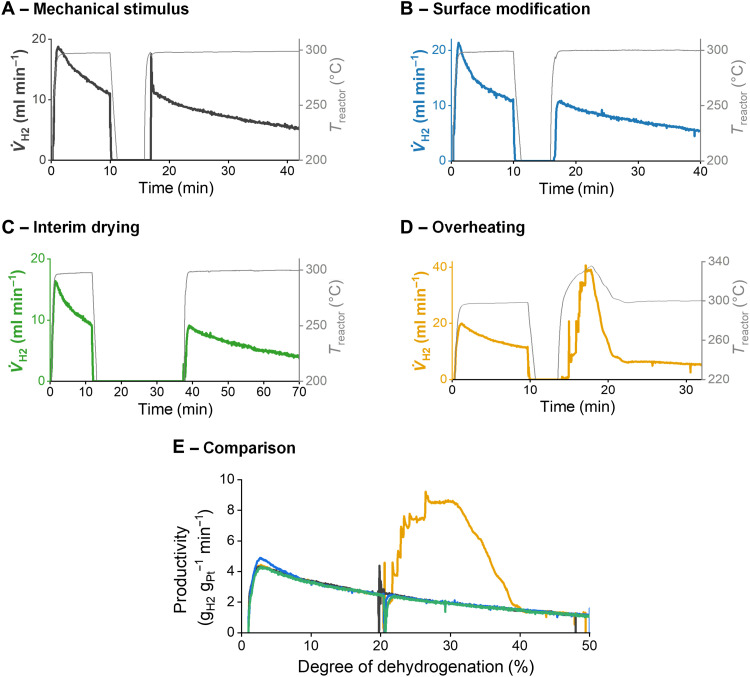
Comparison of different activation techniques in batch dehydrogenations of 1 g of H18-DBT with eight pellets of 0.3 wt% of Pt/alumina catalyst in a glass tube at 300°C with interim cooling at 20% DoDH and reheating. (**A**) Mechanical stimulus with thermocouple after reheating to 300°C, with 129.8 mg of catalyst (see also movie S4). (**B**) Flourosilane-modified catalyst with 0.3 wt% of loading of trichloro-perfluorooctyl silane, with 130.9 mg of catalyst. (**C**) Drying of wet catalyst in a basket with argon (20 ml min^−1^) at 200°C for 10 min between cooling and reheating, with 113.8 mg of catalyst. (**D**) Overheating to 342°C and subsequent cooling to 300°C, with 136.3 mg of catalyst (see also movie S5). (**E**) Comparing catalyst productivies of (A) to (D) over DoDH.

The third column (Fig. 4C) displays the active states of the untreated and fluorosilane-modified catalyst during dehydrogenation. Many more bubbles were observed on the fluorosilane-modified catalyst. While reheating the system after cooling, the treated catalyst started to form bubbles at temperatures as low as 260°C (see also Fig. 5B). This demonstrates that the observed inhibition of the untreated catalyst after a cooling-heating cycle can be avoided by a modification of the surface, as shown in the fourth column (Fig. 4D). The hydrogen release is as high as in the first run, so full reactivation was achieved.

### Other activation techniques

In addition to mechanical activation (Fig. 5A) and catalyst surface modification (Fig. 5B), we introduce two other reactivation approaches that are particularly suited for technical gas generation applications.

#### Drying of the catalyst

Intermediate removal of the liquid phase can be a straightforward strategy to reinsert gas bubbles into the porous systems and recover activity. In Fig. 5C, the catalyst was dried in between cooling and reheating cycles by removing the pellets from the liquid and subjecting them to a hot argon stream. After reimmersion and reheating, the catalyst recovered its full activity. This adapted drying procedure proved also successful for retaining full activity in demonstrator-scale LOHC dehydrogenation reactors (in our case, 1.4 kg of catalyst pellet mass) in repeated start-up and shut-down cycles. We note that catalyst drying is a suitable way to avoid fully liquid-filled pores and to bring the catalyst into a state that resembles the virgin catalyst utilization.

#### Overheating

When heating our eight-pellet test set in H18-DBT beyond our typical reaction temperature of 300°C, the first pellet activated at 315°C, the second at 319°C, the third at 325°C, four other pellets at 328°C, and the last one at 332°C (see movie S5). The activation of each pellet came along with a sudden increase of the observed hydrogen productivity (Fig. 5D). An increased temperature facilitates bubble nucleation in several ways: (i) The dehydrogenation reaction rate increases exponentially, thus raising the local supersaturation of dissolved hydrogen in the pores, and (ii) the surface tension of the organic liquid decreases with increasing temperature, decreasing the energy barrier for the formation of new gaseous interfaces. The interplay of these effects can facilitate nucleation events that finally lead to the full activation of the previously inhibited catalyst pellets. To rationalize the different reactivation temperatures, we state that the first reactivated pellet forms bubbles that lower the supersaturation in the bulk. Thus, the concentration gradient from the pores of inhibited pellets to the bulk becomes steeper, and the diffusional flow of dissolved hydrogen in inhibited pellets becomes higher, lowering the supersaturation in the pores. Reactivation by overheating can be easily implemented in technical reactors if the heat source for dehydrogenation is capable for overheating, and the thermal stability of the applied organic liquid and catalyst is high enough.

In conclusion, we show that nucleation of gas bubbles in the catalyst pores during heterogeneously catalyzed gas generation reactions can represent a so-far overlooked performance-limiting step. Our study reveals that critical inhibition of bubble formation occurs if the porous catalyst network is entirely filled with a well-wetting liquid (i.e., contact angle close to zero).

Without bubble nucleation, the formed hydrogen leads to a fast supersaturation of the fluid phase around the active site, and only hydrogen diffusion in the liquid phase enables dehydrogenation. Such a scenario has been reproducibly found, for example, in shut-down/start-up cycles of the catalytic hydrogen release reaction from the LOHC compound H18-DBT. For this specific reaction, we have demonstrated that the hydrogen release rate in a bubble nucleation–inhibited state is over 50 times lower compared to the hydrogen release rate in a bubbling state at otherwise identical reaction conditions. This marked rate increase is due to an enhanced mass transfer by an oscillating reaction regime, in which the bubble formation and release processes induce convective gas and liquid flows in the pores.

We present four different techniques to overcome this performance limitation: (i) A mechanical stimulus can provide the activation energy for bubble nucleation; (ii) oleophobic surfaces can be introduced into the reactor or onto the surface of the active catalyst pellet to facilitate bubble nucleation; (iii) drying of the catalyst during cool down can prevent the complete filling of the catalyst pellet with liquid, and this avoids nucleation inhibition; and (iv) overheating of the catalyst pellets can trigger bubble formation in cases where the thermal stability of the reactants and of the applied catalyst is sufficient. For the example of catalytic hydrogen release from H18-DBT, we could demonstrate that all four measures allow for full activation of the nucleation-inhibited catalyst (Fig. 5E). Furthermore, bubble nucleation is facilitated under operation conditions, enhancing the rate of the dehydrogenation reaction, i.e., high temperature, low pressure, and low DoDH.

The gained knowledge is of general relevance for reactions that produce large amounts of gaseous products from liquid reaction systems with the help of heterogeneous catalysts or electrocatalysts. These reactions are highly relevant for the emerging green hydrogen economy. Advanced surface designs and innovative dynamic operation procedures promise higher productivities, when bubble nucleation is taken into account as a potential performance-limiting step, as described here.

## MATERIALS AND METHODS

### Dehydrogenation experiments

**Fig. 6.**
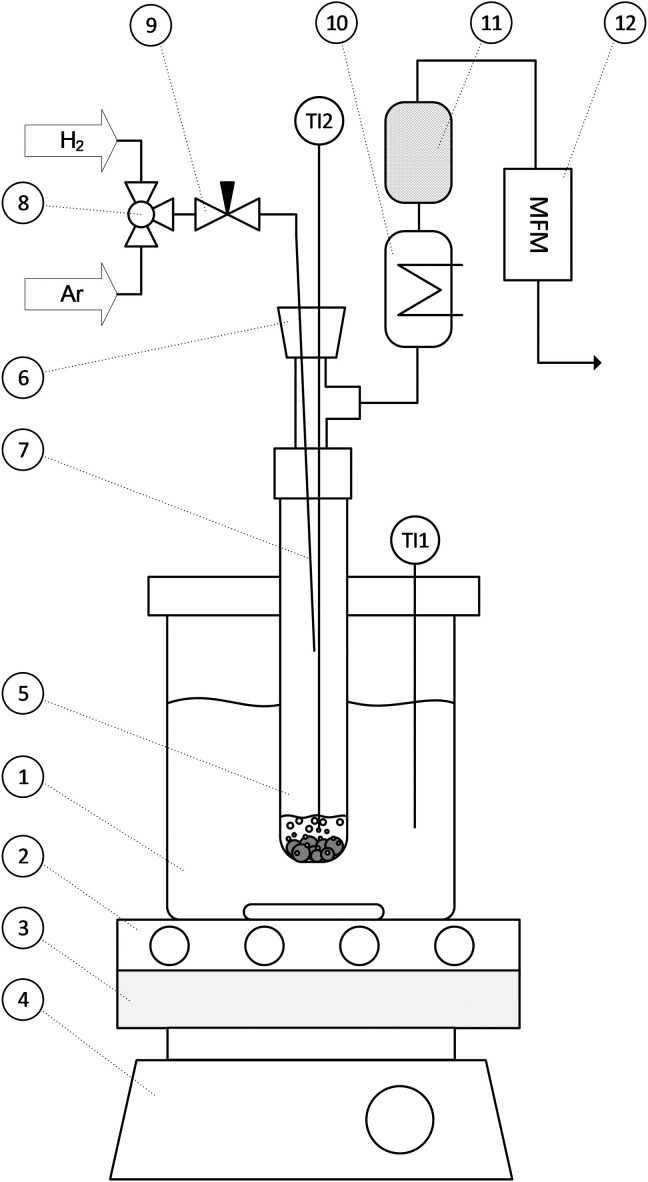
Reactor setup for batch dehydrogenation experiments. With (1) oil bath, (2) heating plate, (3) heat insulation, (4) magnetic stirrer, (5) glass tube reactor with LOHC and eight pellets, (6) septum, (7) gas inlet, (8) three-way valve, (9) needle valve, (10) condenser, (11) activated carbon filter, (12) MFM, (TI1) thermocouple for oil bath, and (TI2) thermocouple for reactor.

The dehydrogenation setup is shown in Fig. 6. It consisted of a 160-mm-long glass tube reactor, which was heated in a stirred cylindrical oil bath. Argon and hydrogen lines were connected to the reactor to flush the inside. A thermocouple measured the reactor bulk temperature and was also used to mechanically reactivate the catalyst pellets. The released hydrogen passed through a condenser, an activated carbon filter, and a mass flow meter (MFM; Vögtlin Instruments GmbH, Switzerland) to quantify the volumetric flow of hydrogen. The catalyst was filmed with a VW-600C camera on a VW-9000D controller and a VW-Z2 objective (Keyence Corporation, Japan) at frame rates of up to 1000 frames/s (fps) for optical analysis.

#### Standard dehydrogenation procedure

The reactor tube was prepared with eight randomly selected pellets of the spherical 0.3 wt% of Pt/alumina eggshell catalyst EleMax-102D (Clariant, Switzerland), which varied in weight due to their size distribution (111 to 136 mg). After flushing with argon for 5 min, 1 g of H18-DBT (Hydrogeneous Technologies, Germany) was added, and the system was fully flushed with hydrogen. To start the reaction, the tube was placed into the oil bath. The temperature of the oil bath was held at 300.5° ± 0.5°C. To reach this temperature rapidly, the oil bath was overheated by 4°C before the cold tube was inserted. After a DoDH of 20% was reached, the reactor was taken out of the oil bath and cooled down below 100°C. Then, the reactor was reheated in the oil bath identically to the first heating. The experiment was deliberately stopped after reaching a DoDH of 50%. At the beginning of each heating and reheating step, the MFM indicated a sudden volume flow because of the thermal expansion of the gas in the tube. Therefore, the first 16 s of the measurement after insertion of the tube was neglected, as the volume flow was not caused by the hydrogen release. Furthermore, liquid samples of the product were taken and analyzed by ^1^H–nuclear magnetic resonance spectroscopy to calculate the DoDH as previously reported (*35*). These values were compared to the DoDH values calculated from MFM data. All experiments showed less than 1% deviation in the DoDH between both methods.

#### Variations of the standard procedure

In the reference dehydrogenation experiment (Fig. 1D), the reactor was not cooled and reheated. The temperature was kept at 300 °C throughout the whole experiment.

In the mechanical reactivation experiments (Figs. 1E and 5A), the inhibited pellets were manually pushed against each other or pressed against a fixed resistance using a thermocouple. All pellets were reactivated by the mechanical stimuli.

The forced liquid infiltration experiments (Fig. 1F) were carried out in the following way: The catalyst pellets were placed in a glass tube, which was evacuated to 10^−3^ mbar and flushed with hydrogen three times to remove inert gases from the pores. The pellets were then heated to 120°C at 10^−3^ mbar for 30 min to remove adsorbed water. Three grams of H18-DBT with a DoDH of 20% was added at 80°C and 10^−3^ mbar. The elevated temperature was used to reduce the liquid viscosity and to allow rapid wetting of the pores. The pellets were cooled to room temperature after 30 min, and the vacuum was held for further 3 days. The tube was then filled with hydrogen, and the eight infiltrated pellets were used for dehydrogenation. In this experiment, LOHC with 20% DoDH was used as a starting material to use comparable conditions to the pellets inhibited by cooling and reheating. The mechanical reactivation was carried out in an identical manner compared to the previous experiment.

The preparation of a surface-modified dehydrogenation catalyst (Fig. 5B) was carried out in the following manner: 500 mg of the technical catalyst and a vial containing 7 mg of trichloro-perfluorooctyl silane (Sigma-Aldrich, USA) were placed in a 500-ml screw top flask, which was equipped with a holder for the vial. The flask was evacuated to 8 mbar for 7 min while shaking at 50 rpm to reach a homogeneous loading of the moving pellets. The pressure was then slowly increased to ambient over 2 min. The vial and the pellets were weighed for calculation of the loading. The pellets were then heated in an argon atmosphere at 8 mbar and 200°C to remove the unannealed silanes. Before the dehydrogenation experiments, the prepared tube with pellets and H18-DBT were evacuated to 8 mbar, the pellets were immersed, and then the pressure was increased to ambient again to infiltrate the pores and to avoid floating of the oleophobic pellets.

The drying of the liquid-filled catalyst pellets (Fig. 5C) was carried out in situ. The catalyst pellets were placed in a self-built catalyst basket made of a V4A wire mesh, which was movable inside the tube. After the pellets cooled down from dehydrogenation, the basket and the thermocouple were lifted out of the liquid (distance to liquid of 8 cm). The tube was then locally heated with two heat guns to 200°C for 10 min in an argon stream of 20 ml min^−1^.The pellets were then cooled down and reimmersed into the liquid, and the tube was reheated in the oil bath.

For the overheating experiment (Fig. 5D), the oil bath was set to 345°C when reheating. After all the pellets reactivated, the oil bath was cooled to 300°C again, to realize comparable catalyst productivities.

The critical reactivation energy was measured using only one pellet. After reheating at a DoDH of 20%, a stiff wire (*d* = 1 mm) weighing 1.15 g was dropped onto the nucleation-inhibited pellet from different heights ranging from 0.2 to 0.8 mm and recorded at 1000 fps. The velocity of the wire was analyzed in the last three frames before impact. When the pellet reactivated, the reactor was cooled and reheated to yield an inhibited state for the next repetition from a different height.

#### Calculations

DoDH values were calculated using Eq. 2. The mass of the starting material *m*_LOHC_ was 1 g, its initial DoDH(*t*_0_) was 1% (unless stated differently), and the volumetric hydrogen stream $\${\dot{V}}_{H2}\$$was measured with an MFM. The productivity *P* was calculated using Eq. 3. The mass of the eight randomly picked pellets *m*_cat_ varied because of their size distribution and ranged from 110.8 to 136.3 mg. The Pt loading of the catalyst *w*_Pt, cat_ was 0.3 wt%

$$DoDH(t)=DoDH(t_{0})+\frac{n_{H2,released}(t)}{n_{H2,LOHC}(t_{0})}=DoDH(t_{0})+\frac{\Sigma_{0}^{t}{\dot{V}}_{H2}(t)\cdot \frac{\rho_{H2}}{M_{H2}}}{\frac{m_{LOHC}}{M_{LOHC}}\cdot 9\cdot (1-DoDH(t_{0}))}$$ (2)

$$P(t)=\frac{{\dot{V}}_{H2}(t)\cdot \rho_{H2}}{m_{cat}\cdot w_{Pt,cat}}$$ (3)

### Pore characterization of the catalyst

#### Sample pretreatment

Before the argon physisorption and mercury intrusion experiments, the samples were degassed overnight at a temperature of 150°C under a turbomolecular pump vacuum. Argon adsorption-desorption isotherms were recorded at liquid argon temperature (87.3 K) using an Autosorb iQ automatic volumetric adsorption analyzer from Quantachrome (Boynton Beach, FL, USA). Argon for analysis with a purity of ≥99.9999% (6.0) was purchased from Air Liquide Deutschland (Düsseldorf, Germany). The pore size distribution of up to a pore size of 90 nm was determined by applying the equilibrium model of nonlocal density functional theory to the desorption branch of the isotherm. Cylindrical, siliceous pores were assumed.

Mercury intrusion and extrusion experiments on the samples were performed at room temperature at pressures ranging up to 415,000 kPa using a Quantachrome PoreMaster 60 instrument. The mercury used for analysis was purchased from Gesellschaft für Metallrecycling (Leipzig, Germany) with a purity of ≥99.9995% (5.5 N).

The pore size distribution was calculated from the pressure-volume data by application of the Washburn equation. The surface tension of mercury was assumed to be 480 mN m^−1^. A contact angle of 140° was chosen.

### Surface analysis

The wetting experiments under reaction conditions (300°C, hydrogen atmosphere) were carried out in a quartz glass cuvette with an aluminum lid. Each sample was clamped to a holder to fix it centrally, and the cuvette was flushed with hydrogen and heated with two heat guns from both sides. A thermocouple measured the inside temperature. When 300° ± 3°C was reached, the droplet of H18-DBT was placed on the surface. Images were taken with the mentioned VW-9000D system and analyzed with the software DSA4 (Krüss GmbH, Germany). The dense samples were a 0.65-mm-thick alumina plate (CeramTec GmbH, Germany), a 1-mm-thick PTFE sheet (Hightechflon GmbH & Co. KG, Germany), and an alumina plate, which was modified with trichloro-perfluorooctyl silane in the same manner as described for the catalyst. The sheets were cut into 6-mm by 3-mm platelets to fit in the holder. For the wetting of porous samples, catalyst support pellets (γ-alumina) with a diameter of 3.5 mm (Clariant, Switzerland) were used, of which the top third was sanded down to obtain a flat surface. The surface modification of the flattened support pellets with trichloro-perfluorooctyl silane was conducted in the same way as described for the catalyst. Because the untreated support wets excellently, the experiment was recorded with 1000 fps to analyze images directly after contact.

To determine the surface free energy of a solid using the model of Owens, Wendt, Rabel, and Kaelble (OWRK), test liquids with known polar and dispersive surface tensions are used to measure contact angles on the solid material. The contact angles of water (Milli-Q), diiodomethane, decane, and toluene (all Sigma-Aldrich, USA) droplets were measured on dense alumina substrates and PTFE, having volumes of 5, 1, 2.5, and 3 μl, respectively. In the case of PTFE and perfluoro-modified alumina, ethanol (Sigma-Aldrich) was added as a test liquid due to low surface energies. A contact angle goniometer DSA 100 with the corresponding software DSA4 (Krüss GmbH, Germany) was used to conduct these measurements under ambient conditions. The surface morphology of the samples plays a crucial role when measuring contact angles. Depending on the hydrophobicity and the surface roughness, the contact angle is changed. The two possible scenarios are described as Cassie-Baxter (*36*) and Wenzel (*37*) wetting. Therefore, roughness effects, even occurring at nominally flat surfaces, are a cause of error in the determination of the surface free energy.

For measuring radial energy-dispersive x-ray depth profiles of platinum and fluorine, an untreated and perfluoro-modified catalyst pellet was split in half. The elemental composition was measured on the exterior surface of the top half and on the inner cross section of the bottom half with a Noran System Six (Thermo Fisher Scientific Inc., USA) using an acceleration voltage of 5 kV.

The surface tension of H18-DBT at high temperatures was measured in the same cuvette as described for the wetting experiments under reaction conditions. The cuvette was flushed with hydrogen, and after reaching and keeping the measurement temperature for 3 min, hanging drops from a cannula were recorded and analyzed with the software DSA4.

#### Calculations

The surface free energy with its relative dispersive and polar parts was calculated using the model of OWRK (*38*, *39*). In general, the relationship between the surface tension of a solid and a liquid is described by Young’s Eq. 4 (*40*)

$$\gamma_{s}=\gamma_{sl}+\gamma_{\mathrm{lg}}\cdot \cos\theta$$ (4)

where γ_s_ is the surface free energy of the solid; γ_sl_ and γ_lg_ are the interfacial energies of solid-liquid and liquid-gas, respectively; and θ is the measured contact angle of the test liquid. Fowkes (*41*) suggested in a theoretical consideration of attractive forces to split the surface tension to take into account the presence of different molecular interactions at surfaces. Thus, the surface free energy and surface tension can be written as a sum of dispersive and polar contributions, γ^d^ and γ^p^, respectively, arising from dipole-dipole and hydrogen bonding interactions in the latter case

$$\gamma =\gamma^{d}+\gamma^{\;p}$$ (5)

OWRK developed a linear Eq. 6 based on these two approaches, whereas the slope and the interception are depicted as the square root of the polar and the dispersive summands of the surface free energy of the solid surface (*38*, *39*)

$$\frac{\gamma_{\mathrm{lg}}(1+\cos\theta )}{2\sqrt{\gamma_{\mathrm{lg}}^{d}}}=\sqrt{\gamma_{s}^{\;p}}\sqrt{\frac{\gamma_{\mathrm{lg}}^{\;p}}{\gamma_{\mathrm{lg}}^{d}}}+\sqrt{\gamma_{s}^{d}}$$ (6)

## Supplementary Materials

### This PDF file includes:

Supplementary text Figs. S1 to S8 References
