# Supplementary material for: Nucleation as a rate-determining step in catalytic gas generation reactions from liquid phase systems
Source: Sci Adv. 2022 Nov 16;8(46):eade3262. doi: 10.1126/sciadv.ade3262 (PMC9668311; doi:10.1126/sciadv.ade3262)
Supplement: Supplementary file 1 — Supplementary text Figs. S1 to S8 References [file sciadv.ade3262_sm.pdf]

Science Advances
AAAS

Supplementary Materials for

Nucleation as a rate-determining step in catalytic gas generation reactions from liquid phase systems

Thomas Solymosi *et al.*

Corresponding author: Peter Wasserscheid, p.wasserscheid@fz-juelich.de

Sci. Adv. **8**, eade3262 (2022)
DOI: 10.1126/sciadv.ade3262

The PDF file includes:

Legends for movies S1 to S5
Supplementary text
Figs. S1 to S8
References

Other Supplementary Material for this manuscript includes the following:

Movies S1 to S5

Supplementary Movies

Movie S1: Different states of catalyst at 300 °C and mechanical activation (1000 fps). The active state is characterized by vigorous bubble formation whereas the catalyst does not form bubbles in the inhibited state. The catalyst can be brought into the active state by a mechanical stimulus.

Movie S2: Bubble nucleation shortly after mechanical activation of the nucleation-inhibited pellet. The growing motion of the light areas on the pellet surface from left to right indicates that the gas bubble grows within the oversaturated pore system and displaces the liquid. At some positions of the pellet (presumably wide pore mouths) bubbles emerge into the bulk liquid as soon as the light area reached these positions. This reproducible observation suggests that a nucleation-inhibited pellet is liquid filled and lacks a gas nucleus.

Movie S3: The inhibited catalyst produces an oversaturated liquid with dissolved hydrogen and bubbles nucleate on the surface of an inert PTFE cube next to it. Bubble nucleation is enhanced on the PTFE surface due to its lower surface energy and thus, lower wetting abilities.

Movie S4: Mechanical activation of the inhibited catalyst after cooling and reheating. After the mechanical stimulus the bubble nucleus grows throughout the porous pellet. Each pellet must be individually reactivated. The bubble motion of a reactivated neighboring pellet is not sufficient to activate a pellet. Full activity is reached after every pellet was reactivated

Movie S5: Activation of the inhibited catalyst by overheating up to a reactor temperature of 335 °C with subsequent cooling to 300 °C. The pellets reactivate at different temperatures above 300 °C. After reactivation of all pellets and cooling to 300 °C, full activity was achieved.

Supplementary Results

High temperature surface tension of H18-DBT

Figure **S1** shows the surface tension of H18-DBT at different temperatures under H_2 atmosphere. With increasing temperature, the surface tension decreases in a linear way. The surface tension at 300 °C, which was used in equation 1, was linearly extrapolated and is 12.3 mN m^{-1}.

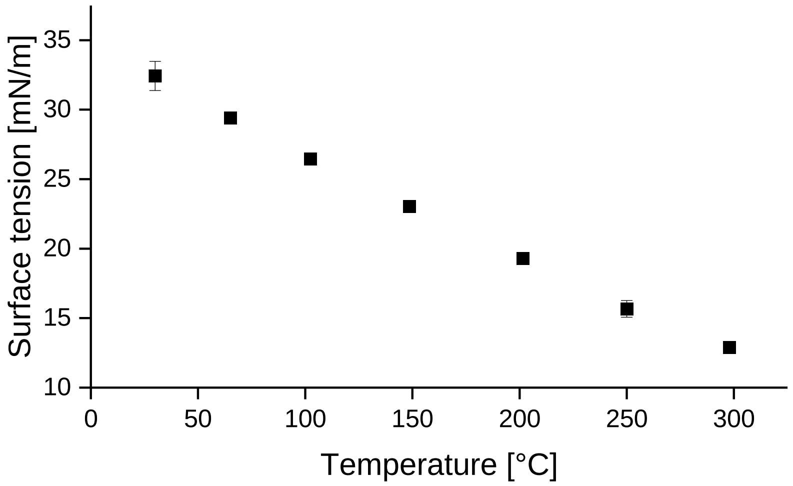

Fig. S1: Surface tension of H18-DBT dependent on temperature measured with pendant drop under H_2 atmosphere.

Pore size distribution and mode pore size of the catalyst

The Ar 87K adsorption-desorption isotherms (Fig. **S2**) reveal pore condensation hysteresis indicating the presence of meso/macropores but does not exhibit a plateau at high relative pressure. This indicates that, while the mesopore system could, the macropore system could not be completely filled with liquid Ar. Accordingly, the calculated NLDFT pore size distribution (Fig. **S3**) does not reflect the whole intraparticular pore system, but still allowed one to calculate the mesopore size distribution which is centered around a mode pore diameter of ca. 30 nm (mode pore radius of ca. 15 nm). A full pore size/volume distribution could be obtained by combining argon adsorption with mercury intrusion, which reflects the complete meso/macropore network of the catalyst (Fig. **S4**). The specific surface area amounts to ca. 26 $m^2 g^{-1}$ which was obtained by the application of the BET theory to the Ar 87K adsorption isotherm in the relative pressure range of 0.05-0.35.

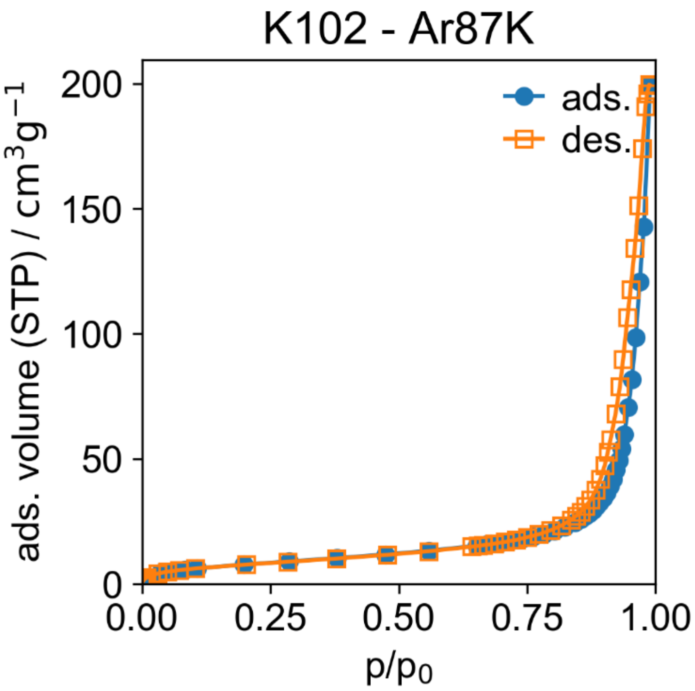

Fig. S2: Argon 87K adsorption-desorption isotherms show structural characteristics of the catalyst material.

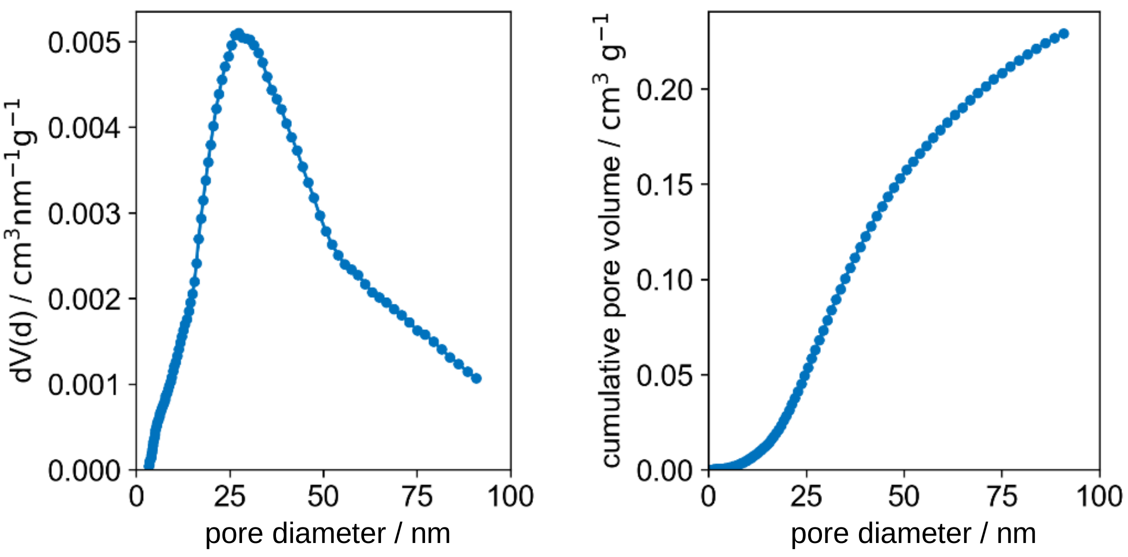

Fig. S3: Differential (left) and cumulative (right) NLDFT pore volume distributions over pore diameter.

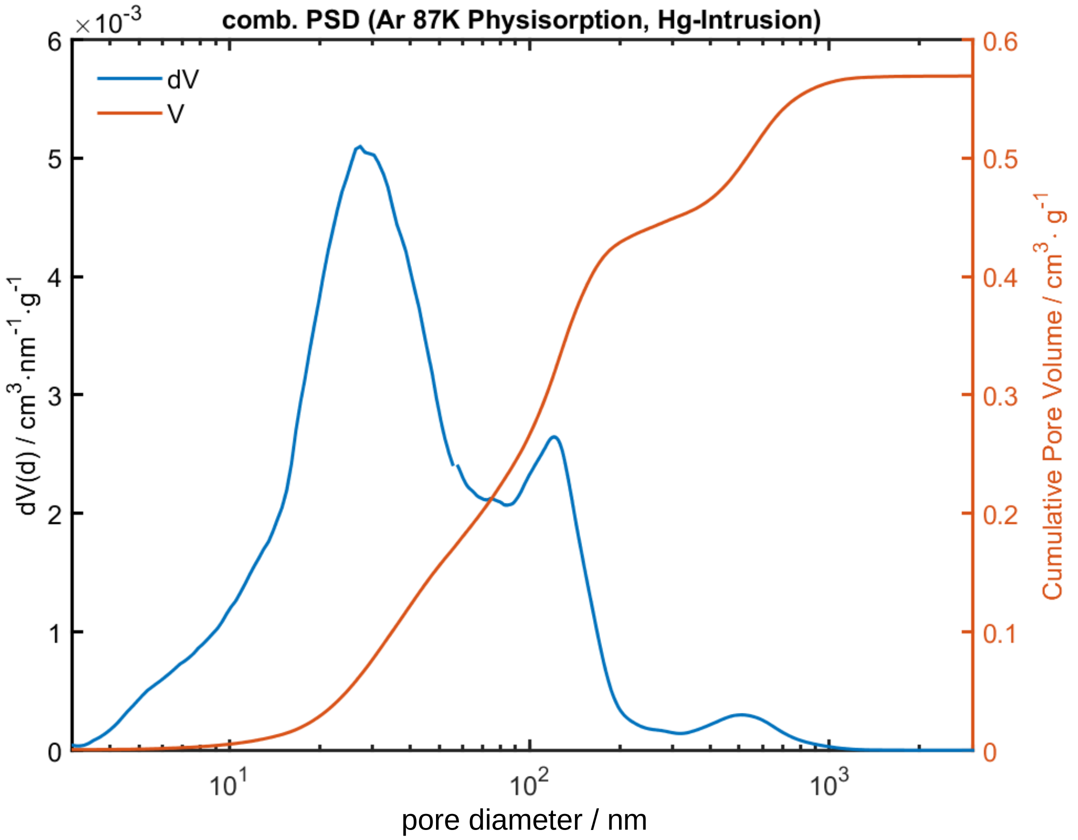

Fig. S4: Differential (left axis) and cumulative (right axis) pore volume distributions over the whole pore size range combined from Ar 87K physisorption and Hg intrusion experiments.

Surface Free Energy (SFE) of alumina, PTFE and perfluoro-modified alumina

The contact angle measurements at ambient conditions reveal a successful perfluoro-modification of the alumina samples. The contact angle of water on the perfluoro silane-modified alumina increased from low values (40°) to large values (102°) within the range of PTFE, indicating the presence of a hydrophobic surface chemistry (Fig. **S5A**). For calculations of the surface energy of a solid, the OWRK-model was used, as described above. The linear fit allows determination of the polar and dispersive surface energies calculated from the slope and intercept, respectively (**S5B**). A steeper linear regression indicates a more pronounced polar effect on γ_s, as for pure alumina samples, whereas in case of PTFE the slope is almost zero, indicating that the surface energy is caused entirely by dispersive interactions. The perfluoro-modified alumina shows a flat slope indicating a dominant effect of dispersive interactions, as expected for the fluorinated surface functionalization with perfluorooctyl silane. However, small contributions of polar interactions demonstrate that the underlying alumina substrate still partially contributes to the surface energy. The overall surface energy of the perfluoro-modified alumina sample is reduced as compared to plain alumina and shows values close to the PTFE reference (**S5C**). This modification results in a decreased substrate wettability of the samples.

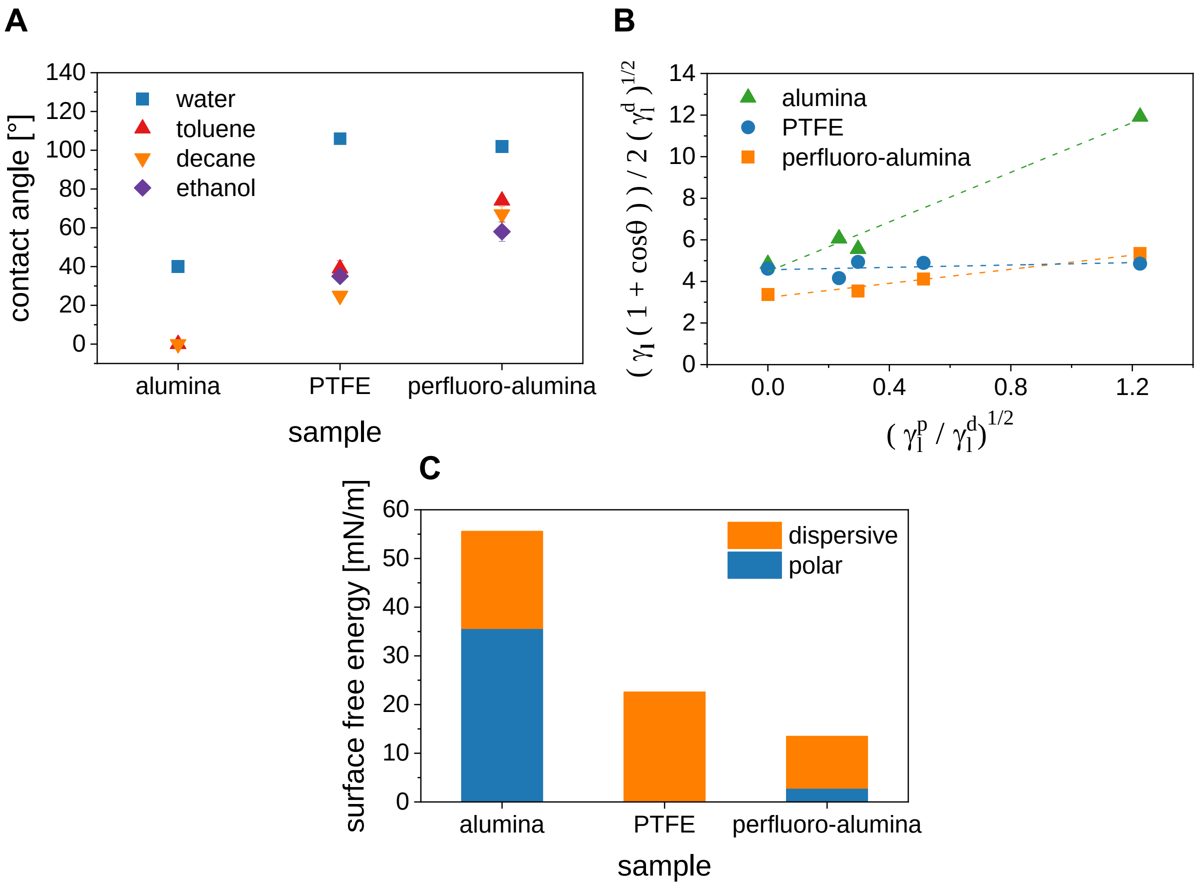

Fig. S5: Surface properties for different substrates: alumina, PTFE and perfluoro-modified alumina. (**A**) Contact angles on three model substrates measured with different test liquids: water, toluene, decan and ethanol. (**B**) A linear regression, calculated using the OWRK model, is used to determine the slope and intercept that are directly linked to the polar and dispersive part of the surface energy, according to equation S5. (**C**) Comparison of surface energies of alumina, PTFE and perfluoro-modified alumina with its different contributions calculated according to the OWRK model.

EDX characterization of the perfluoro-modified catalyst

Figure **S6** displays the radial EDX-profiles of the platinum and fluorine mass fractions of the untreated catalyst (left) and of the perfluoro-modified catalyst over the depth of a half pellet. It is evident that the silane was deposited mainly on the exterior surface and only to a small extent within the outer 80 μm shell. Since the platinum eggshell is approximately 300 μm deep, the wetting of the inner part of the pellet is not affected by the surface modification. This explains why the hydrogen release productivity in the dehydrogenation experiment is comparable to the untreated catalyst in the active state.

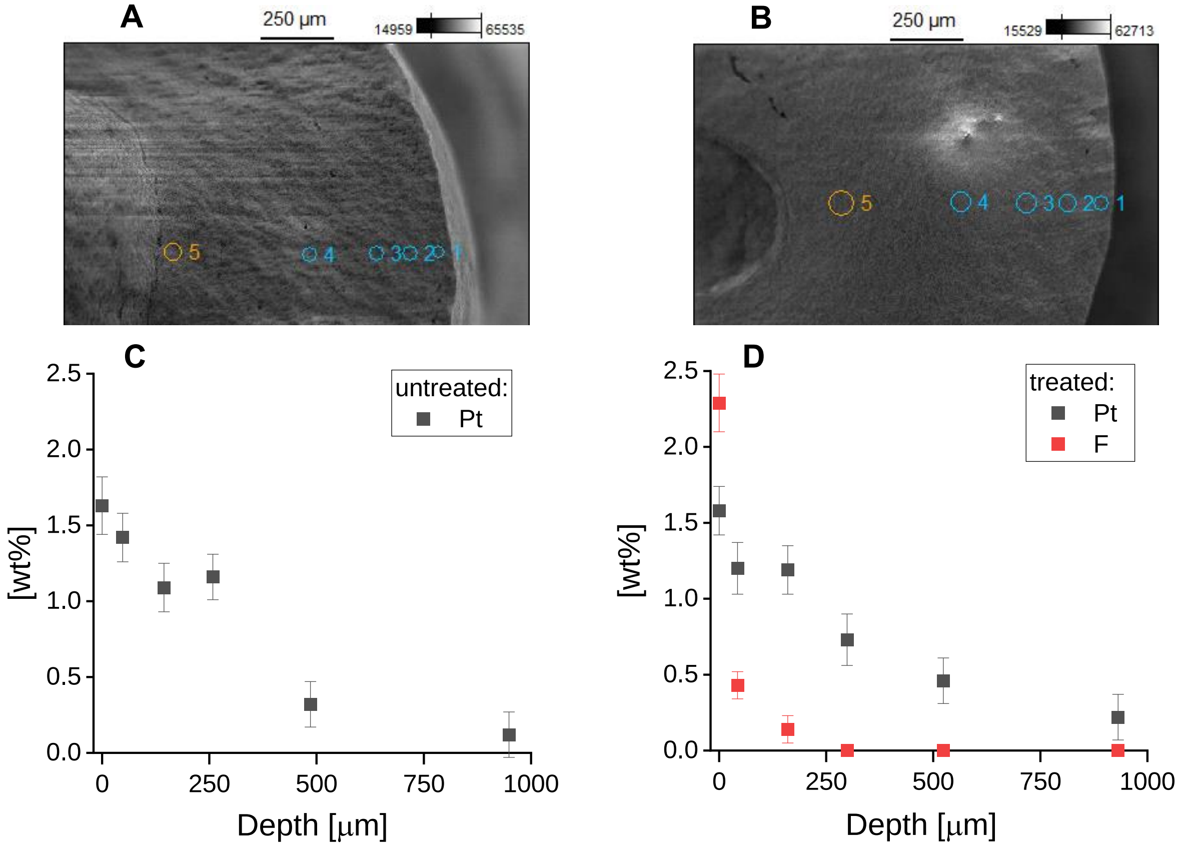

Fig. S6: EDX measurements of the untreated (left) and the perfluoro-modified (right) catalyst pellet that were split in half. Images and positions for EDX-measurement of the half untreated (**A**) and perfluoro-modified (**B**) pellet. Radial depth profiles of platinum and fluorine content for the untreated (**C**) and perfluoro-modified (**D**) pellet.

Dehydrogenation experiment with many PTFE cubes

To examine the influence of oleophobic surfaces on the hydrogen release rate, the standard dehydrogenation procedure was conducted in addition with a high number (32) of PTFE cubes inside the H18-DBT. Figure **S7A** shows that after reheating the preferred bubble nucleation on the cubes increased the hydrogen release of the inhibited catalyst to 5% of the active state compared to <1.8% without PTFE cubes. All the bubbles nucleated and grew on the PTFE surface (**S7B**). As soon as the pellets were mechanically activated, the nucleation and bubble growth on the PTFE surface stopped (**S7C**). Because the reactivated pellets form many bubbles offering a large liquid-gas-interface, the supersaturation of hydrogen is decreased directly at the site of formation. So, although the PTFE cubes increase the hydrogen release in an inhibited state at high supersaturation, they do not improve an active catalyst system.

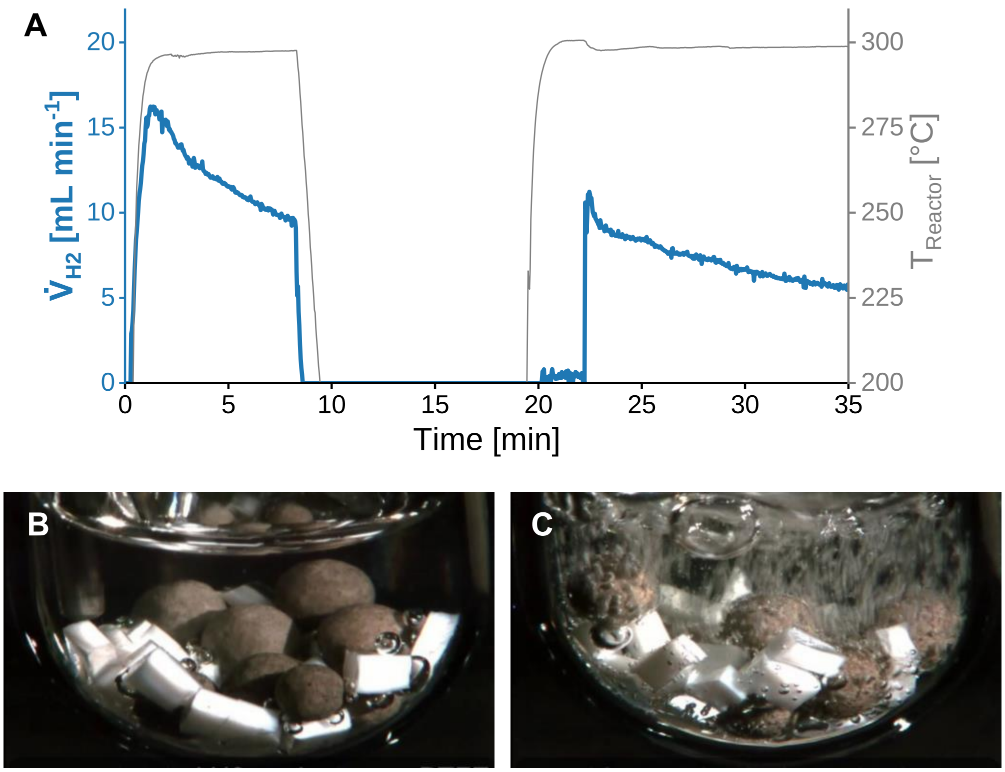

Fig. S7: Enhanced nucleation on PTFE surfaces at oversaturation of dissolved H2. (**A**) Standard dehydrogenation experiment with 32 PTFE cubes and mechanical activation 2 min after reheating (114.3 mg catalyst, 215 mg PTFE cubes). (**B**) Bubbles nucleate on PTFE cubes in oversaturated liquid when catalyst is in its inhibited state. (**C**) After mechanical activation bubbles on PTFE cubes stop growing.

Bubble formation model in supersaturated liquid-gas mixtures

In order to assess the role of the mechanical activation we compare the energy required to nucleate a bubble of size R with the energy provided by the mechanical activation. We determined the required activation energy by dropping a metal wire from different heights onto a nucleation-inhibited pellet at 300 °C. While energies of ≤ 1.0 mJ were not sufficient to trigger nucleation, all impacts with a critical energy of ≥ 1.7 mJ activated the pellet. Assuming that this critical mechanical activation energy E was homogeneously distributed across the pellet (d_{pellet} = 3 mm), the available energy ε for a bubble of size R is

$$\varepsilon \equiv E\frac{V_b}{V_P} = \frac{E}{V_P}\frac{4}{3}\pi R^3, \tag{S1}$$

where V_b is the volume of the bubble and V_P is the volume of the pellet. In order to calculate the energy required to nucleate the bubble, we assume to have a solution of H18-DBT and H_2 in contact with a gas of H_2 at pressure P and temperature T. Since we choose P, T and the number N of H_2 molecules in the system as independent variables, the relevant thermodynamic potential is the Gibbs free energy which in the case of a homogeneous solution reads

$$G_1(N,P) = N\mu_L(P,\rho_1)\,, \tag{S2}$$

where ρ_1 is the number density of the H_2 molecules and μ_L is their chemical potential at given pressure and density. We restrict ourselves to an isothermal bubble formation and drop the dependence on the temperature to ease the notation.

After the bubble formation, N_G molecules have "left" the H18-DBT solution and have formed a bubble of radius R. Accordingly, the free energy reads

$$G_2(N,P) = 4\pi\gamma R^2 + (N - N_G)\mu_L(P,\rho_2) + N_G\mu_G(P_b)\,, \tag{S3}$$

where γ is the surface tension, $N - N_G$ is the number of H_2 molecules dissolved in the H18-DBT, ρ_2 is their number density and $\mu_G(P)$ is the chemical potential of the H_2 which we assume to be like an ideal gas

$$\mu_G(P_b) = k_B T\,\ln\left(\frac{P_b}{k_B T}\right). \tag{S4}$$

Here, k_B is the Boltzmann constant.

$$P_b = P + \frac{2\gamma}{R} \tag{S5}$$

is the pressure inside the bubble due to both atmospheric pressure P and Laplace pressure $2\gamma/R$. We introduce the change in the chemical potential in the solution

$$\Delta\mu_L \equiv \mu_L(P, \rho_2) - \mu_L(P, \rho_1) \tag{S6}$$

as well as the difference in the chemical potential between the molecules in the gas bubble and those in solution

$$\Delta\mu_G \equiv k_B T \ln\left(\frac{P_b}{k_B T}\right) - \mu_L(P, \rho_2)\,. \tag{S7}$$

Hence, using eqs. S6 and S7 the free energy difference reads

$$\Delta G \equiv G_2 - G_1 \;=\; 4\pi\gamma\, R^2 \;+\; N\Delta\mu_L \;+\; N_G\Delta\mu_G\;. \tag{S8}$$

In this expression we can identify three mechanisms:

1. $4\pi\gamma R^2$ is the increase in the free energy due to the creation of a fluid interface.

2. $N\,\Delta\mu_L$ is the change in the free energy in the bulk of the H18-DBT solution due to the decrease in the density of the dissolved H_2 molecules.

3. $N_G\Delta\mu_G$ is the change in the free energy due to the molecules that formed the bubble.

In order to compute the last expression, we need to write down the dependence of the number of molecules in the gas bubble N_G and the chemical potential in the liquid μ_L on N, P, T. For what concerns the number of molecules in the gas bubble N_G, we determine it by the equation of state of an ideal gas

$$N_G = \frac{4\pi R^3}{3k_B T}\left[P + \frac{2\gamma}{R}\right]. \tag{S9}$$

Concerning the chemical potential μ_L, in typical experimental situations we can assume that the relation between ρ and P is linear. In particular this occurs when the density of H_2 in the H18-DBT well exceeds the saturation density $\bar{\rho}$ *(42)*. In this regime we assume the chemical potential at large P and for $\rho/\bar{\rho} > 1$

$$\mu_L(P, \rho) = \alpha \ln(\rho), \tag{S10}$$

where α refers to the H_2 effective chemical potential when suspended in H18-DBT solution. In the case of no interactions (ideal gas) we have $\alpha = k_B T$, while for attractive interactions we expect $\alpha < k_B T$ and for the repulsive case $\alpha > k_B T$.

In our case the following applies:

1. $N_G \ll N$: the number of molecules forming the bubble is much smaller than the total number of molecules.

2. $\frac{N_G}{N} \gg \frac{\Delta V}{V_1}$: the relative variation of the number of molecules of gas in solution is larger than the associated change of the volume of the solution.

Accordingly, expanding $\Delta\mu_L$ for $N_G \ll$ N leads to

$$\Delta\mu_L \simeq -\alpha \frac{N_G}{N}. \tag{S11}$$

Substituting the expressions for $\Delta\mu_L$ and $\Delta\mu_G$ in eq. S8 leads to

$$\Delta G \simeq 4\pi\gamma R^2 \left(1 - \frac{2}{3}\left(\frac{\alpha}{k_B T} - \ln\left(\frac{P + 2\gamma/R}{k_B T \rho_1^{\alpha/k_B T}}\right)\right)\right) - \frac{4}{3}\pi R^3 P \left(\frac{\alpha}{k_B T} - \ln\left(\frac{P + 2\gamma/R}{k_B T \rho_1^{\alpha/k_B t}}\right)\right). \tag{S12}$$

It is insightful to rewrite the following expression as a function of the supersaturation defined as $\rho_1/\bar{\rho}$ with $\bar{\rho}$ being the saturation density at equilibrium. In order to do so we recall that at equilibrium the pressure of the outside gas matches the atmospheric pressure

$$P = k_B T \rho_{gas}, \tag{S13}$$

with ρ_{gas} being the number density inside the gas phase over the liquid and the chemical potential of the H_2 dissolved in the H18-DBT solution equals that of the H_2 dissolved in the gas phase

$$\alpha \ln(\bar{\rho}) = k_B T \ln(\rho_{gas}). \tag{S14}$$

To simplify the notation, we introduce $R_0 = \frac{\gamma}{P}$ and $\Gamma = \frac{\alpha}{k_B T}\left(1 + \ln\left(\frac{\rho_1}{\bar{\rho}}\right)\right) - \ln\left(1 + 2\frac{R_0}{R}\right)$. Finally, the free energy ΔG can be rewritten as

$$\Delta G \simeq \frac{4}{3}\pi R_0^3 P \frac{R^3}{R_0^3}\left[(3-2\Gamma)\frac{R_0}{R} - \Gamma\right]. \quad \text{(S15)}$$

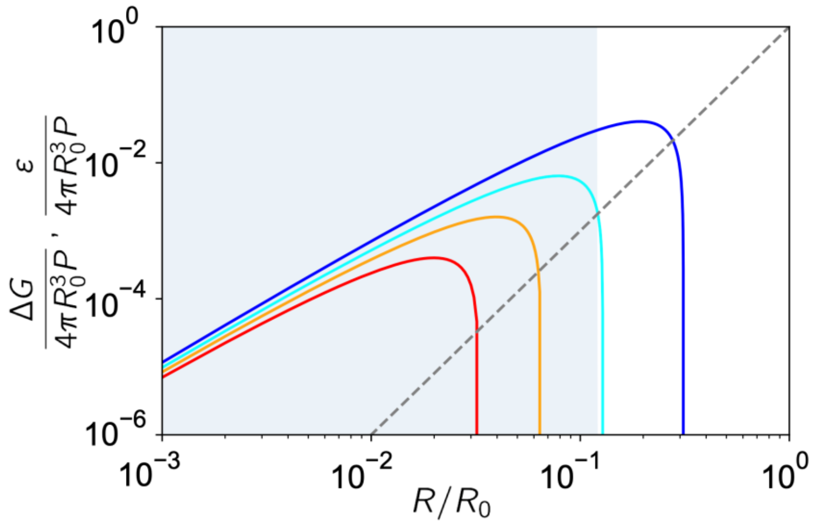

Fig. S8: Normalized energy induced by mechanical activation $\boldsymbol{\varepsilon}$ (grey dashed line) as a function of the normalized bubble radius $\boldsymbol{R/R_0}$. ε corresponds to the maximum available energy to possibly nucleate bubbles. Here, we use the mechanical activation energy $E \simeq 1.7 \cdot 10^{-3}$ Nm and a pellet volume of $V_P \simeq 4 \cdot 10^{-9}$ m^3 in agreement with our measurements. The solid lines show the normalized free energy ΔG for three values of the supersaturation $\rho_1/\bar{\rho} = 10{,}25{,}50{,}100$ (blue, cyan, orange, and red, respectively). The shaded area marks bubbles with a radius smaller than the mode pore size, $R_{pore} \simeq 15$ nm for $R_0 \simeq 120$ nm, which is what we expect for H18-DBT at atmospheric pressure. The values of the supersaturation used in this figure are in agreement with those reported in the literature (*25*). Our simple model explains why a threshold in the mechanical activation energy has to be reached to nucleate stable bubbles: If the energy is too small, the minimum stable bubble size increases beyond the typical pore size which thus renders this event unlikely.

Figure **S8** shows the comparison of the normalized free energy difference ΔG (see Eq. S15), and the normalized energy provided by the mechanical activation ε (see Eq. S1) as function of the normalized radius of the bubble R/R_0. For H18-DBT in contact with a gas at atmospheric pressure and 300 °C the surface tension is $\gamma \simeq 0.012$ N/m which leads to $R_0 \simeq 120$ nm. Accordingly, Figure S8 shows that for values of the supersaturation $\rho/\bar{\rho} \in [10{,}100]$ the energy provided by the mechanical activation is comparable to that required by the nucleation process. Note that local negative pressure fluctuations during the impact causes the supersaturation to abruptly increase as the saturation concentration $\bar{\rho}(P)$ decreases, facilitating nucleation. Moreover, the size of the nucleated bubbles is $R \lesssim 15$ nm and, as shown in the figure, these bubbles are on the "descending" branch of the free energy, namely, once nucleated, these bubbles will grow. Accordingly, this explains the sensitivity of the onset of bubbling on the intensity of the mechanical activation reported in this manuscript: In order to nucleate stable bubbles in a pore, a minimal mechanical activation energy is needed. For lower energies, the minimum stable bubble size increases beyond the typical pore size which thus renders this event unlikely. This implies that for larger pore sizes a lower mechanical activation energy is sufficient.

REFERENCES AND NOTES

1. A. Jess, P. Wasserscheid, *Chemical Technology: From Principles to Products* (John Wiley & Sons, ed. 2, 2020).

2. P. Preuster, C. Papp, P. Wasserscheid, Liquid organic hydrogen carriers (LOHCs): Toward a hydrogen-free hydrogen economy. *Acc. Chem. Res.* **50**, 74–85 (2017).

3. M. Niermann, S. Timmerberg, S. Drünert, M. Kaltschmitt, Liquid organic hydrogen carriers and alternatives for international transport of renewable hydrogen. *Renew. Sustain. Energy Rev.* **135**, 110171 (2021).

4. F. Caupin, E. Herbert, Cavitation in water: A review. *C. R. Phys.* **7**, 1000–1017 (2006).

5. S. Jones, G. Evans, K. Galvin, Bubble nucleation from gas cavities—A review. *Adv. Colloid Interface Sci.* **80**, 27–50 (1999).

6. P. Wilt, Nucleation rates and bubble stability in water-carbon dioxide solutions. *J. Colloid Interface Sci.* **112**, 530–538 (1986).

7. H. J. Maris, Introduction to the physics of nucleation. *C. R. Phys.* **7**, 946–958 (2006).

8. A. Angulo, P. van der Linde, H. Gardeniers, M. Modestino, D. F. Rivas, Influence of bubbles on the energy conversion efficiency of electrochemical reactors. *Joule* **4**, 555–579 (2020).

9. M. Maier, K. Smith, J. Dodwell, G. Hinds, P. R. Shearing, D. J. L. Brett, Mass transport in PEM water electrolysers: A review. *Int. J. Hydrogen Energy* **47**, 30–56 (2022).

10. R. J. Thorne, C. Sommerseth, A. P. Ratvik, S. Rørvik, E. Sandnes, L. P. Lossius, H. Linga, A. M. Svensson, Bubble evolution and anode surface properties in aluminium electrolysis. *J. Electrochem. Soc.* **162**, E104 (2015).

11. Z. Yuan, K. Zuo, C. Cao, S. Chen, W. Chuai, Z. Guo, The whole process, bubble dynamic analysis in two-phase transport of the passive miniature direct methanol fuel cells. *J. Power Sources* **416**, 9–20 (2019).

12. P. Lv, H. Le The, J. Eijkel, A. Van den Berg, X. Zhang, D. Lohse, Growth and detachment of oxygen bubbles induced by gold-catalyzed decomposition of hydrogen peroxide. *J. Phys. Chem. C* **121**, 20769–20776 (2017).

13. G. Liang, I. Mudawar, Review of pool boiling enhancement by surface modification. *Int. J. Heat Mass Transfer* **128**, 892–933 (2019).

14. J. Chen, S. Ahmad, J. Cai, H. Liu, K. T. Lau, J. Zhao, Latest progress on nanotechnology aided boiling heat transfer enhancement: A review. *Energy* **215**, 119114 (2021).

15. J. Mo, Z. Kang, S. T. Retterer, D. A. Cullen, T. J. Toops, J. B. Green, M. M. Mench, F.-Y. Zhang, Discovery of true electrochemical reactions for ultrahigh catalyst mass activity in water splitting. *Sci. Adv.* **2**, e1600690 (2016).

16. Z. Kang, J. Mo, G. Yang, S. T. Retterer, D. A. Cullen, T. J. Toops, J. B. Green Jr., M. M. Mench, F.-Y. Zhang, Investigation of thin/well-tunable liquid/gas diffusion layers exhibiting superior multifunctional performance in low-temperature electrolytic water splitting. *Energ. Environ. Sci.* **10**, 166–175 (2017).

17. Z. Kang, G. Yang, J. Mo, S. Yu, D. A. Cullen, S. T. Retterer, T. J. Toops, M. P. Brady, G. Bender, B. S. Pivovar, J. B. Green, F.-Y. Zhang, Developing titanium micro/nano porous layers on planar thin/tunable LGDLs for high-efficiency hydrogen production. *Int. J. Hydrogen Energy* **43**, 14618–14628 (2018).

18. C. Brussieux, P. Viers, H. Roustan, M. Rakib, Controlled electrochemical gas bubble release from electrodes entirely and partially covered with hydrophobic materials. *Electrochim. Acta* **56**, 7194–7201 (2011).

19. H.-J. Butt, K. Graf, M. Kappl, *Physics and Chemistry of Interfaces* (John Wiley & Sons, 2013).

20. L. B. Datsevich, Alternating motion of liquid in catalyst pores in a liquid/liquid–gas reaction with heat or gas production. *Catal. Today* **79-80**, 341–348 (2003).

21. L. B. Datsevich, Oscillation theory: Part 4. Some dynamic peculiarities of motion in catalyst pores. *Appl. Catal. Gen.* **294**, 22–33 (2005).

22. L. B. Datsevich, Some theoretical aspects of catalyst behaviour in a catalyst particle at liquid (liquid–gas) reactions with gas production: Oscillation motion in the catalyst pores. *Appl. Catal. Gen.* **247**, 101–111 (2003).

23. H. Jorschick, P. Preuster, S. Dürr, A. Seidel, K. Müller, A. Bösmann, P. Wasserscheid, Hydrogen storage using a hot pressure swing reactor. *Energ. Environ. Sci.* **10**, 1652–1659 (2017).

24. S. Dürr, S. Zilm, M. Geißelbrecht, K. Müller, P. Preuster, A. Bösmann, P. Wasserscheid, Experimental determination of the hydrogenation/dehydrogenation-equilibrium of the LOHC system H0/H18-dibenzyltoluene. *Int. J. Hydrogen Energy* **46**, 32583–32594 (2021).

25. S. R. German, M. A. Edwards, Q. Chen, Y. Liu, L. Luo, H. S. White, Electrochemistry of single nanobubbles. Estimating the critical size of bubble-forming nuclei for gas-evolving electrode reactions. *Faraday Discuss.* **193**, 223–240 (2016).

26. S. R. German, M. A. Edwards, H. Ren, H. S. White, Critical nuclei size, rate, and activation energy of H_2 gas nucleation. *J. Am. Chem. Soc.* **140**, 4047–4053 (2018).

27. G. Edwards, L. Evans, S. Hamann, Nucleation of ice by mechanical shock. *Nature* **223**, 390–391 (1969).

28. R. Wylie, The freezing of supercooled water in glass. *Proc. Phys. Soc. B* **66**, 241–254 (1953).

29. C. Marcolli, Ice nucleation triggered by negative pressure. *Sci. Rep.* **7**, 1–8 (2017).

30. J. W. Mullin, K. D. Raven, Nucleation in agitated solutions. *Nature* **190**, 251–251 (1961).

31. A. Sivakumar, S. M. B. Dhas, Shock-wave-induced nucleation leading to crystallization in water. *J. Appl. Cryst.* **52**, 1016–1021 (2019).

32. R. B. Dean, The formation of bubbles. *J. Appl. Phys.* **15**, 446–451 (1944).

33. L. Léal, M. Miscevic, P. Lavieille, M. Amokrane, F. Pigache, F. Topin, B. Nogarède, L. Tadrist, An overview of heat transfer enhancement methods and new perspectives: Focus on active methods using electroactive materials. *Int. J. Heat Mass Transfer* **61**, 505–524 (2013).

34. R. N. Wenzel, Surface roughness and contact angle. *J. Phys. Chem.* **53**, 1466–1467 (1949).

35. G. Do, P. Preuster, R. Aslam, A. Bösmann, K. Müller, W. Arlt, P. Wasserscheid, Hydrogenation of the liquid organic hydrogen carrier compound dibenzyltoluene—Reaction pathway determination by ^{1}H NMR spectroscopy. *React. Chem. Eng.* **1**, 313–320 (2016).

36. A. Cassie, S. Baxter, Wettability of porous surfaces. *Trans. Faraday Society* **40**, 546–551 (1944).

37. R. N. Wenzel, Resistance of solid surfaces to wetting by water. *Ind. Eng. Chem. Res.* **28**, 988–994 (1936).

38. D. K. Owens, R. Wendt, Estimation of the surface free energy of polymers. *J. Appl. Polym. Sci.* **13**, 1741–1747 (1969).

39. D. Kaelble, Dispersion-polar surface tension properties of organic solids. *J. Adhes.* **2**, 66–81 (1970).

40. T. Young III, An essay on the cohesion of fluids. *Philos. Trans. R. Soc. London* **95**, 65–87 (1805).

41. F. M. Fowkes, Attractive forces at interfaces. *Ind. Eng. Chem. Res.* **56**, 40–52 (1964).

42. R. Aslam, K. Müller, M. Müller, M. Koch, P. Wasserscheid, W. Arlt, Measurement of hydrogen solubility in potential liquid organic hydrogen carriers. *J. Chem. Eng. Data* **61**, 643–649 (2016).
